# Data Collection from Buried Sensor Nodes by Means of an Unmanned Aerial Vehicle

**DOI:** 10.3390/s22155926

**Published:** 2022-08-08

**Authors:** Christophe Cariou, Laure Moiroux-Arvis, François Pinet, Jean-Pierre Chanet

**Affiliations:** INRAE, UR TSCF, University of Clermont Auvergne, 9 Av. Blaise Pascal CS 20085, F-63178 Aubiere, France

**Keywords:** environmental monitoring, Internet of Underground Things, Wireless Underground Sensor Networks, Unmanned Aerial Vehicle, evolutionary algorithms, LoRa, ZigBee

## Abstract

The development of Wireless Underground Sensor Networks (WUSNs) is a recent research axis based on sensor nodes buried a few dozen centimeters deep. The communication ranges are, however, highly reduced due to the high attenuation of electromagnetic waves in soil, leading to issues of data collection. This paper proposes to embed a data collector on an Unmanned Aerial Vehicle (UAV) coming close to each buried sensor node. The whole system was developed (sensor nodes, data collector, gateway) and experimentations were carried out in real conditions. In hovering mode, the measurements on the RSSI levels with respect to the position of the UAV highlight the interest in maintaining a high altitude when the UAV is far from the node. In dynamic mode, the experimental results demonstrate the feasibility of carrying out the data collection task while the UAV is moving. The speed of the UAV has, however, to be adapted to the required time to collect the data. In the case of numerous buried sensor nodes, evolutionary algorithms are implemented to plan the trajectory of the UAV optimally. To the best of our knowledge, this paper is the first one that reports experiment results combining WUSN and UAV technologies.

## 1. Introduction

In the face of global climate change, monitoring the health and degradations of ecosystems is becoming of crucial importance to understand phenomena, anticipate changes and engage appropriate actions [1]. A first approach consists of analyzing high-resolution satellite images to measure visible evolutions of some phenomena at a large scale (e.g., melting of mountain glaciers [2], reduction of surface waters [3], changes in land use and land cover [4]). A second one consists of deploying sensor nodes directly in the environment, with the aim to perform precise measurements on specific points and obtain information which is impossible to access from an aerial system.

The data collection of sensor nodes installed in wide and isolated areas (e.g., natural zones, mountainous regions, agricultural lands) may be a challenging task. In fact, the communication links between the sensor nodes and the closest gateway can not be always possible, for example due to devices out of range, perturbations or problems of network coverage. The telephony base stations (GSM) can moreover be inaccessible. In addition, a strong constraint to be considered is the minimization of the energy consumption of the nodes. The objective is in fact generally to position the nodes for ideally several years without battery replacement, that requires to limit as much as possible the energy spent to communicate. Finally, although rarely aborded in the literature, the protection of the sensor nodes against potential damages (e.g., animals, climatic events, vandalism, passage of vehicles) is also to be considered with attention.

**Figure 1:**
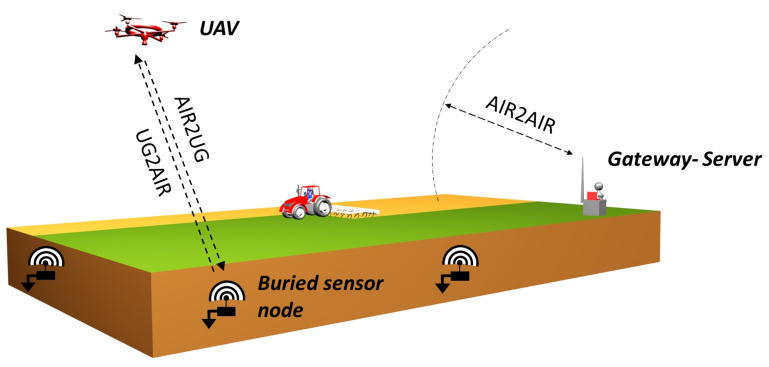
Environmental monitoring system is based on a set of sensor nodes distributed underground. Periodically, the UAV collects the data by following an optimized flight trajectory. The data are delivered to the remote gateway at the end of the mission.

To meet these different expectations, we propose in this paper to combine the recent advances offered by the Wireless Underground Sensor Networks (WUSNs) with the potential provided by the Unmanned Aerial Vehicles (UAVs), see the illustration in Figure 1. In this scheme, each sensor node, composed of the sensor, the battery, the electronic components, the radio module, and the antenna, is fully buried at a few dozen of centimeters deep. That enables them to protect themselves against numerous external damages and does not impact the aboveground activities. The UAV is used for its part as a data collector and acts as an interface between the sensor nodes and the remote gateway. By coming close to each sensor node, the UAV enables moreover to limit the transmit power of the radio modules and, therefore, the energy consumption of the nodes. During the flight, the collected data frames are first memorized on the UAV. They are next unloaded on the gateway at the end of the mission. In the case of numerous sensor nodes to be visited, the trajectory of the UAV can be advantageously optimized to minimize its flight time.

Research on buried sensor nodes is relatively recent and has rapidly led to the concepts of Wireless Underground Sensor Networks (WUSNs) and the Internet of Underground Things (IoUT) [5,6,7,8]. Typical targeted applications are smart irrigation, detection of pollution near rivers, and detection of landslides [9,10,11]. This new paradigm is, however, strongly constrained by the fact that the radio electromagnetic signals are highly attenuated in the soil, about 20–300 times worse than in the air [12]. The propagation distance of the signals is therefore highly reduced underground. The problem is that the attenuation of the radio signals through the soil is dependent on numerous factors as the soil properties (volumetric water content, percentages of sand, silt, and clay, bulk density, temperature) and depends on the configuration of the node (e.g., burial depth, operating frequency, transmit power, antenna orientation) [13,14,15]. Typically, a wet soil (dependent on weather, topography, and environment) highly attenuates the radio signal (e.g., about +20 dB compared to dry soil [16]), as well as high temperature, clay soil, and high bulk density. The soil parameters are unfortunately spatially heterogeneous, and the volumetric water content (VWC) highly variable. The VWC also impacts the antenna radiation pattern [12], and the antenna orientation has to be carefully considered (0 to 100% packet error rate) [16]. As a result, the Underground to Underground (UG2UG) communication link between two buried sensor nodes remains difficult to establish and reaches only a few meters in favorable conditions [16]. However, while the development of a WUSN based on multi-hop operations seems difficult to achieve, our last work [17] highlighted that the Underground to Aboveground (UG2AG) communication link can reach more than 275 m under certain conditions (868-MHz radio modules with the technology LoRa [18], transmit power of +14 dBm/25 mW, 15 cm burial depth, aboveground receiving antenna at 2 m height). Similarly, [19] presented successful UG2AG transmissions in LoRaWAN [20] for sensor nodes buried from 10 cm to 50 cm at a distance of 27 m.

Meanwhile, recent advances in UAVs have led to a progressive increase in their autonomy, payload, and maneuverability. That has enabled us to open a large panel of new applications [21,22]. In particular, UAVs are becoming more and more integrated aboveground. Wireless Sensor Networks (WSNs) to improve connectivity, coverage, and quality of service [23], with the aim to use them as high-altitude aerial base stations, communication relays, or data collectors [24,25,26,27,28,29,30]. The problem of data collection from a UAV is mainly addressed in the literature in terms of optimal trajectory planning, but also flight time minimization or methods to wake up the nodes. For example, Ref. [31] studied the generation of an optimal trajectory for a UAV passing near several IoT nodes rested on the floor. An interesting approach is proposed to gather some nodes with respect to the strength of the Received Signal Strength Indication (RSSI) levels and thus reduce the number of transmissions. Similarly, Ref. [32] formulated the problem of flight time minimization for a UAV having to collect data of aboveground nodes linearly distributed. The speed of the UAV, which is another essential parameter to be considered in the trajectory planning issue, is adapted with respect to the transmit power of the nodes and the number of data to be collected. The wake-up problem of sensor nodes from a UAV is addressed by [33]. Different devices based on either infrared or radio frequency signals were experimented. Unfortunately, tests were performed only with aboveground components. Results showed the possibility of waking-up some nodes with a delay lower than 80 ms on the condition to have a distance lower than 5 m between the UAV and the node.

Likewise, UAVs could be advantageously used to collect the data of sensor nodes buried in the natural environment. That approach can avoid the installation of numerous and costly gateways and offer the possibility to rapidly and safely cover wide areas (obviously, in the distance and altitude limits allowed by the applicable regulations). However, to the best of our knowledge, the data collection of buried sensor nodes by a UAV has not yet been experimentally investigated in the literature. Only simulation results are presented in [34] where the impacts of different parameters (e.g., UAV altitude, flight time, soil moisture, burial depth) are studied. In this work, 2000 nodes are assumed to be buried and distributed within a field of 20 hectares to collect the data from a stationary UAV using the NB-IoT technology. In addition to the work considering aboveground nodes, particular constraints have to be taken into account for underground systems, such as varying communication ranges, node priorities, and time windows to limit the energy consumption. That requires investigating particular optimization algorithms.

The objective of the study reported in this paper was to experimentally demonstrate the feasibility of retrieving data of buried sensor nodes using a UAV based on LoRa communication. The impact of the UAV’s position and altitude on the strength of the RSSI signals with respect to the position of the sensor node is highlighted from several experimentations. In addition, tests at different speeds are reported highlighting the possibility to collect the data dynamically. In case of several buried sensor nodes, the flight planification issue of the UAV is addressed through evolutionary algorithms. This paper is organized as follows. The materials and methods are presented in Section 2. The experimental setup and results are reported in Section 3. Obtained results are discussed in Section 4. Conclusions and future work are presented respectively in Section 5 and Section 6.

## 2. Materials and Methods

We consider several buried sensor nodes distributed within a wide area. The nearest gateway is assumed to be out of the coverage range of the nodes, leading to no possibility of direct connection to the Internet as it is usually the case in conventional Low Power Wide Area Networks (LPWAN). The network coverage by GSM or WiFi is assumed unavailable. That leads to discarding the solution of embedding a gateway directly on the UAV as there is no possibility of having a continuous connection with the server to transfer the data during the flight. The sensor nodes are buried at a few dozens of centimeters deep. They periodically perform soil parameter measurements which are stored in their internal memories. The challenge consists of retrieving these data while limiting the energy consumption of the nodes.

**Figure 2:**
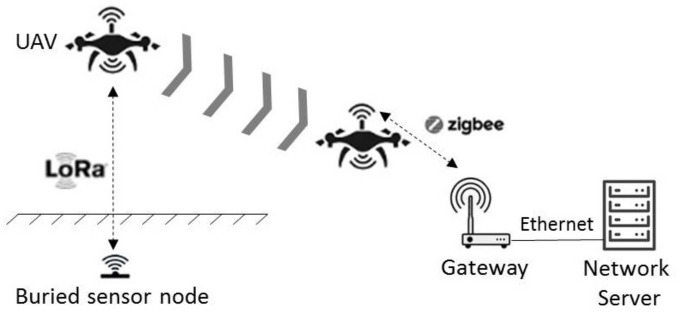
Principle scheme. The Lora technology is used to retrieve the data from the buried sensor nodes, and the Zigbee communication is used to upload the data on the gateway.

The scenario considered is based on a UAV used as a data collector, i.e., embedding an electronic device with communication capabilities with both the sensor nodes and a ground gateway as well as with data collecting and recording capabilities. The UAV flies over the buried sensor nodes following a previously defined flight plan in terms of waypoints, speed, and altitude. The data are first transferred and stored in the internal memory of the collector node embedded on the UAV, to be next, at the end of the mission, sent to the gateway connected to the network server, see the principle scheme in Figure 2. The advantage of such an approach is to bring flexibility with the possibility to place the nodes indifferently in the environment without the need to form a connected network. That discards the constraints of multihops, high communication ranges, and risks of network failures. In addition, the radio parameters for the LoRa communication (e.g., spreading factor) can be tuned to reduce the energy consumption of the nodes. The time during which the UAV can communicate with the buried sensor node during a flight with a high RSSI signal can also be considered. This time determines the maximal number of data that can be transferred. It depends in particular on the communication range, the altitude, and speed of the UAV.

As presented in Figure 2, we propose an architecture based on two radio communication links, namely LoRa and ZigBee. We chose to develop the wireless communication UG2AIR (Underground to Air) between the buried sensor nodes and the collector node embedded in the UAV based on the LoRa technology [18]. This technology offers numerous advantages in the development of WUSNs thanks to its high communication range and its low energy consumption. The quantity of data sent is limited (about 0.29 kbps to 5 kbps) but remains compatible for applications involving low-rate environmental sensors. This technology operates in the ISM frequency band (EU: 868 MHz and 433 MHz, USA: 915 MHz and 433 MHz) and is based on a Chirp Spread Spectrum (CSS) modulation technique that enables to significantly increase both the signal reliability and receiver sensibility while maintaining a low energy consumption. The communication range in LoRa is determined by several configurable parameters such as the bandwidth (BW), the signal output power, and the spreading factor (SF). A high value of SF increases the communication range to the detriment of the data rate, as the signal is transmitted for a longer time, as well as to the detriment of the autonomy as the communication time is longer. The coding rate (CR) is another configurable parameter enabling to apply a set of coding rules on the exchanged signals. A high value of CR offers more protection but increases the time of exchange and decreases the bit rate. In the system developed, following the results of our previous work [17], we used the 868 MHz frequency band with a transmit power tuned at +14 dBm/25 mW, a bandwidth of 125 KHz, and an SF value tuned to 7.

We chose to base the wireless communication AIR2AIR between the data collector and the gateway on the ZigBee protocol [35]. This protocol enables the development of networks with low energy consumption, star, mesh, or tree topologies, as well as a 250 kbps data rate. It is defined by the IEEE 802.15.4 standard dedicated to the Low-Rate Wireless Personal Area Networks (LR-WPAN) having short range and low energy consumption. The ZigBee protocol is based on this standard for the physical layer (PHY) and the medium access control (MAC). The network layer (NMK) and the application layer (APL) are defined and maintained by the enterprise organization known as the Connectivity Standards Alliance (CSA), formerly ZigBee Alliance. The ZigBee network has three kinds of modules, namely *End Device* which is a terminal node (e.g., sensor, actuator), *Router* which routes and transmits the data between the devices and enables one to expand the physical range of a ZigBee network, and *Coordinator* which manages the high-level functions of the network as the authentification and security. In a ZigBee network, there is necessarily a coordinator. ZigBee-Pro is a version of ZigBee that benefits from higher capacities as the routing techniques, the network of jumps, the maximal number of peripherals as well as the security of the network. In the system developed, the ZigBee-Pro network between the collector node and the gateway is configured with a star topology, using the ISM frequency band at 2.4 GHz. We defined the collector node as being the *End Device* and the gateway as the *Coordinator*.

### 2.1. Materials

We developed all the devices presented in Figure 2, namely the sensor nodes, the data collector, and the gateway. This subsection presents these devices.

#### 2.1.1. The Buried Sensor Nodes

**Figure 3:**
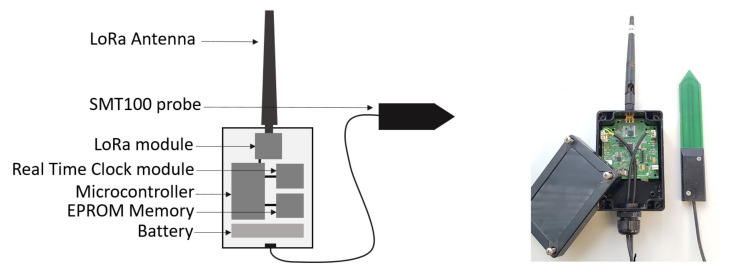
The sensor node is built around an Atmega328 microcontroller, a DS1378 temporal reference, a RFM95W LoRa radio module operating at 868-MHz, and an SMT100 probe. The radio antenna is out of the box and will be directly in contact with the soil.

**Figure 4:**
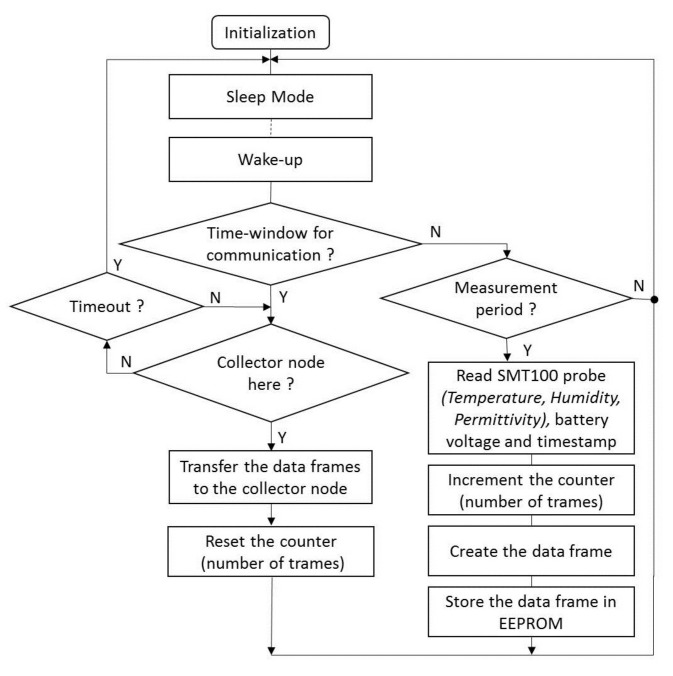
Diagram of the program in a sensor node.

We developed a set of buried sensor nodes enabling, on the one hand, to periodically take and store measurements of soil parameters and, on the other hand, upload the data frames to the collector node embedded on a UAV when it is in proximity. To that end, the sensor node is managed by a control program running on a microcontroller Atmega328-AU 8 MHz (Microchip), see Figure 3. The sensor nodes are powered through a rechargeable lithium battery 3.7 V/8.8 Ah with the aim to be autonomous in energy for several months. The sensor nodes are most of the time in deep-sleep mode. They are in active mode only during the measurements and some communication time-windows. A Truebner SMT100 probe is connected to measure the soil moisture and temperature. The measurements are stored on an EEPROM 8 bit memory with I2C-bus interface. The radio communication is based on the LoRa module RFM95W (HopeRF) including the SX1276 chip of Semtech. To determine the timing for the measurement periods and the communication time windows, a temporal reference DS1378 is implemented; see the diagram of the program in Figure 4. The time-windows enable the sensor node to be ready to communicate with the collector node embedded on the UAV. When a time-window is active, the sensor node puts its radio module in listen mode and waits for a message from the collector node. When the communication is established, the sensor node transfers the data frames to the data collector; see the structure of a data frame in Table 1.

#### 2.1.2. The Data Collector Node and the Gateway

**Figure 5:**
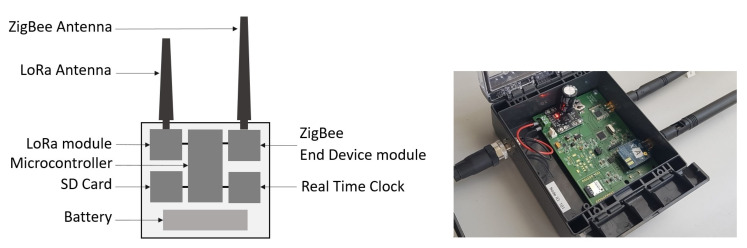
The developed collector node with two antennas. The communication is done in LoRa with the buried sensor nodes and in ZigBee with the gateway (when they are accessible). This collector node is embedded on the UAV.

We developed a collector node including two radio modules: the first one is devoted to the communication in LoRa with the buried sensor nodes, and the second one to the communication in ZigBee with the gateway. The collector node has, therefore, two antennas, a LoRa antenna at 868 MHz and a ZigBee antenna at 2.4 GHz, see Figure 5.

**Figure 6:**
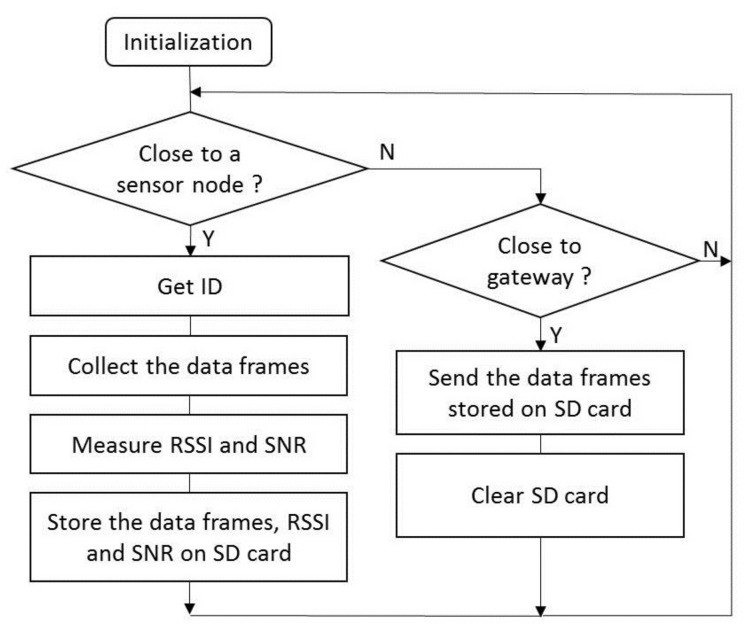
Diagram of the program in the collector node.

The collector node is built around a microcontroller Atmega328-AU (Microchip) running at 8 MHz. It is equipped with a LoRa radio module RFM95W (HopeRF) enabling to communicate with the buried sensor nodes, and a ZigBee radio module Xbee-PRO (Digi International) to exchange with the server through the gateway. The received data frames are stored in an SD card from an SPI bus. The collector node is powered through a rechargeable lithium battery 3.7 V/8.8 Ah. When accessible, the collector node communicates in Zig-Bee with the remote gateway equipped with a ZigBee module. The collector node is the *End Device* and the gateway the *Coordinator*. The diagram of the program in the collector node is presented in Figure 6.

**Figure 7:**
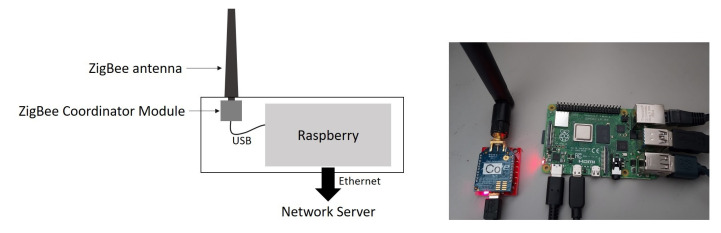
The gateway with the Zigbee communication module is defined as *Coordinator* to retrieve the data from the UAV and put them on the network server.

The gateway is based on a Raspberry Pi 4 board with a 64-bit processor and an 8 Go memory, see Figure 7. It is connected to the network server through an Ethernet link. When the UAV goes back to the base station, the gateway receives and transfers the data frames to the network server. These data are stored on CSV files.

#### 2.1.3. The UAV

**Figure 8:**
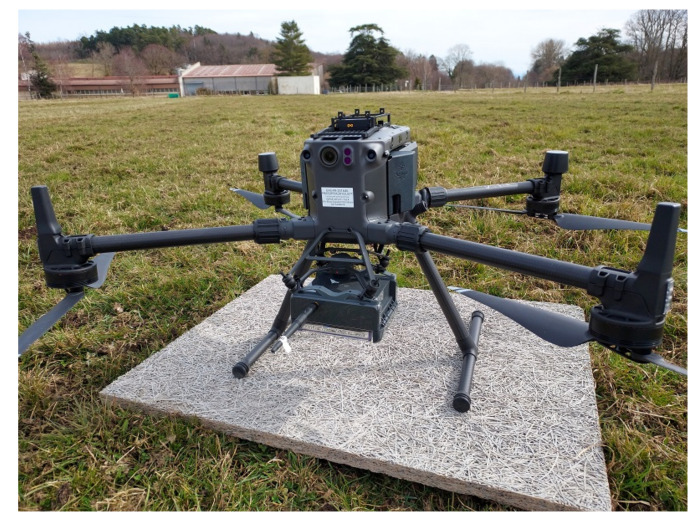
The UAV used for the experimentations (Matrice 300 RTK—DJI) with the collector node attached on the underside.

The UAV used for the experimentations is presented in Figure 8. It is a Matrice 300 RTK (DJI). Its main characteristics are a weight with batteries of about 6.3 kg and a payload of 2.7 kg. Its maximal speed is 23 m/s and its autonomy is about 40 min when the payload is used. In France and outside urban areas, this UAV can be used by a professional pilot either by sight at the maximal altitude of 120 m and a lateral distance of 200 m (this scenario was used to qualify the UG2AIR communication link), or without sight at the maximal altitude of 50 m and a lateral distance of 1000 m (this scenario was used to carry out flights in autonomous mode). The maximal altitude and lateral limits can be increased by derogations.

### 2.2. Methods

#### 2.2.1. Experimental Setup: Hover Flights

**Figure 9:**
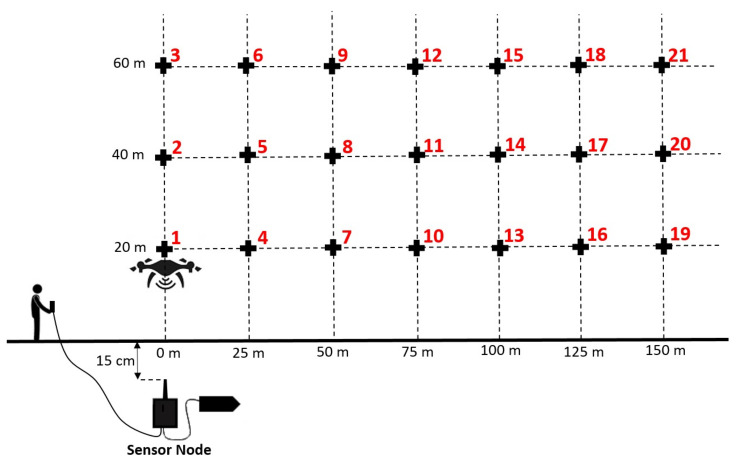
Experimental setup in hovering mode to study the UG2AIR communication link. The UAV is stabilized for several seconds at different altitudes (20, 40, and 60 m) and lateral distances (0 to 150 m by steps of 25 m). At each position, the buried sensor node sends 100 data frames. The collector node measures the RSSI signals and collects the data frames.

To evaluate the quality of the communication link UG2AIR from a sensor node buried at 15 cm deep to the collector node, it was decided to position the UAV in hovering mode at different altitudes (20 m, 40 m, and 60 m) and lateral distances (from 0 m to 150 m by step of 25 m with respect to the position of the buried node), see the operating mode in Figure 9. For each position of the UAV, numbered from 1 to 21, the buried sensor node sent 100 data frames (the composition of a data frame is presented in Table 1). Once the UAV was at its position, a push button connected to the buried sensor node enabled to trigger the transmission of the data frames.

For each measuring point, the UAV was maintained in its position during the time to collect the 100 data frames. The data are received by the collector embedded on the UAV and stored in its internal SD memory card. At each reception of a data frame, the value of the RSSI signal was measured and recorded by the collector node.

#### 2.2.2. Experimental Setup: Dynamic Flights

**Figure 10:**
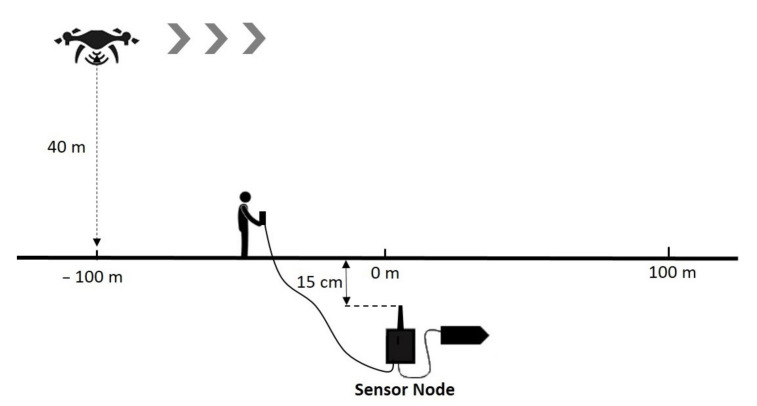
Experimental setup in dynamic mode to study the UG2AIR communication link at different speeds. The UAV flies at the altitude of 40 m. At each reception of a data frame, the collector node measures the level of the RSSI signal.

To evaluate the possibility of retrieving the data frames when the UAV is continuously flying, it was first defined that the UAV had to fly at an altitude of 40 m to have a trajectory free of obstacles. Then, the start was determined at a lateral distance of −100 m from the node that corresponds also to the start of the data transmission of the sensor node. The UAV flies along a line segment of 200 m long, with a speed of successively 4, 6, and 8 m/s, and over the sensor node in the middle of its trajectory, see the operating mode in Figure 10. Similarly, as in stationary experiments, the 100 data frames are received and stored by the collector node embedded in the UAV. The RSSI values are measured and recorded by the collector node.

#### 2.2.3. Optimal Trajectory Planning for the UAV

Once the feasibility will be demonstrated to collect the data frames of a buried sensor node from a collector node embedded in a UAV, the next point to be considered will be the limited flight time of the UAV. In fact, even a high-end UAV cannot generally fly more than forty minutes in favorable conditions (i.e., low payload, air temperature above $20^{{}^{\circ}}$). In case of numerous buried sensor nodes to be visited with limited coverage ranges, the UAV’s trajectory has to be optimized. This problem can be considered a Traveling Salesman Problem (TSP) [36,37]. The time complexity of such a problem will increase with the number of nodes. The total number of possible routes is $(nb_{nodes}-1)!/2$. The calculation of all the possible routes is, therefore, rapidly prohibitive in terms of computational time. For example, 25 nodes lead to about $\left(25-1\right)!/2≃10^{23}$ possible routes. By considering a computer able to calculate 1 billion routes per second, it would take $10^{23}/\left(10^{9}*60*60*24*365\right)=10^{7}$, i.e., 10 million years to calculate all the paths. To find sub-optimal solutions to this problem, we implemented two evolutionary algorithms: a genetic algorithm (GA) and an ant colony optimization (ACO).

- **Genetic Algorithm (GA)**

**Figure 11:**
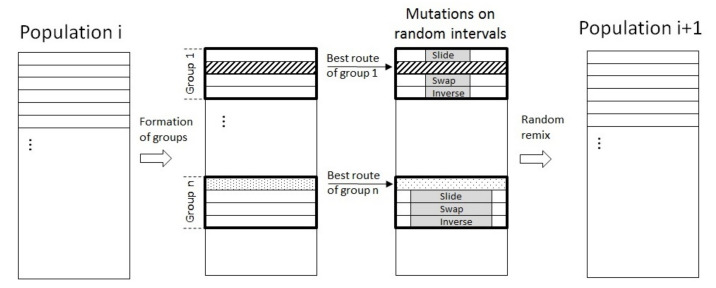
Principle of the genetic algorithm to generate a new population.

The genetic algorithm is an evolutionary algorithm inspired by the evolution theory of Darwin. From an initial population, new generations are successively created with some mutations. The principle of the algorithm proposed in this paper is depicted in Figure 11 and inspired from [38]. The algorithm starts with the creation of a population of one hundred routes of the UAV, randomly chosen and passing above all the buried sensor nodes (waypoints). This population is next divided in groups of four routes: in each group, the best route, i.e., the one with the minimal Euclidian distance, is kept and called the survivor. The other routes of the group are replaced by copies of the survivor and mutations are applied: on a random interval, the node orders are either reversed, shifted, or swapped. All the routes are then randomly remixed to form the new population. This process is repeated until an end condition is satisfied (e.g., the length of the obtained route does not change anymore). The advantage of such an algorithm is to be relatively simple and easily programmable.

- **Ant Colony (ACO)**

**Figure 12:**
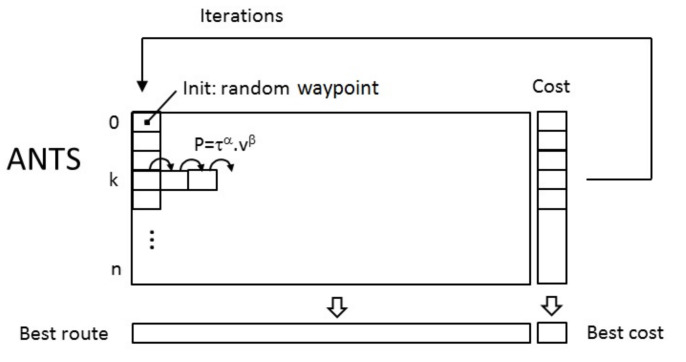
Principle of the ants colony algorithm.

The ant colony algorithm is inspired by the behavior of ants which are able to progressively find the shortest route between their colony and a source of food [39]. This behavior is explained by the fact that each ant deposits an organic substance on the ground called pheromone. The ants will follow the route which has the higher level of pheromone (this substance evaporates in time). At the beginning, the ants explore several routes while depositing pheromones. Progressively, as more a path is followed, more the ants will choose this route, the shortest route is found. The principle of the ACO algorithm used in this paper is presented in Figure 12. At the beginning (left column), the ants are numbered from 1 to n, and are distributed in the different waypoints (n is superior to the number of waypoints *p*). The visibility matrix V(p×p) is calculated (inverse of the distance between two waypoints: constant), as well as the pheromone matrix τ(p×p) (level of pheromone between two waypoints: will vary with time). By considering an ant in a waypoint $w_{k}$, the next waypoint of this ant is determined by the point-to-point multiplication of the line $w_{k}$ of the visibility matrix by the line $w_{k}$ of the pheromone matrix. This gives a probability vector which is presented in the form of cumulative probability varying from 0 to 1. A random value (to give a chance to explore other routes) is compared to the values of this probability vector that determines the next waypoint for this ant. Once all the ants have visited all the waypoints, the best route corresponding to the minimal Euclidian distance is memorized. The pheromone matrix is then updated with this route and the values of the pheromone matrix is decreased with a percentage (evaporation). A new iteration can start.

## 3. Results

### 3.1. Experimental Setup

**Figure 13:**
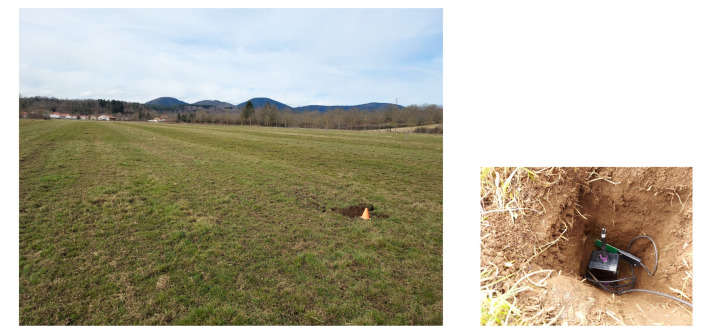
Experimental field (45°42′25.99″ N, 3°00′40.54″ E, 433 m) and a sensor node buried at 15 cm deep. Once the sensor node is installed, some soil is added and compacted by foot.

The tests were carried out in an experimental field of the French National Research Institute for Agriculture, Food and Environment (INRAE), see Figure 13. This field is a flat and free-obstacle pasture with a volcanic soil. The weather conditions were low temperatures (about 10 ${}^{{}^{\circ}}$ C) with a cloudy sky and 57% humidity. A sensor node was buried at 15 cm deep (top of the antenna) and covered with soil. The probe connected to the node was positioned in the hole at the same depth as the radio antenna. The measured values were a soil moisture of 5.47%, a soil temperature of 3.98 °C, and a dielectric permittivity value of 10.38.

To transmit the data frame of 25 bytes presented in Table 1, the time on air in LoRa varies from 45 ms to 958 ms with respect to the value of the spreading factor (SF), see Table 2. We selected the lowest value (SF = 7) to reduce the time on the air at its minimal value and therefore limit the energy consumption of the buried sensor node. The LoRa module on the collector node was tuned on the same SF. A low value of SF leads also to a reduced communication range but that is not issued as the UAV can come close to the buried sensor node. For these tests, the transmit power was maintained at +14 dBm/25 mW, which is the maximal allowed transmit power in Europe in the 868 MHz frequency band.

**Figure 14:**
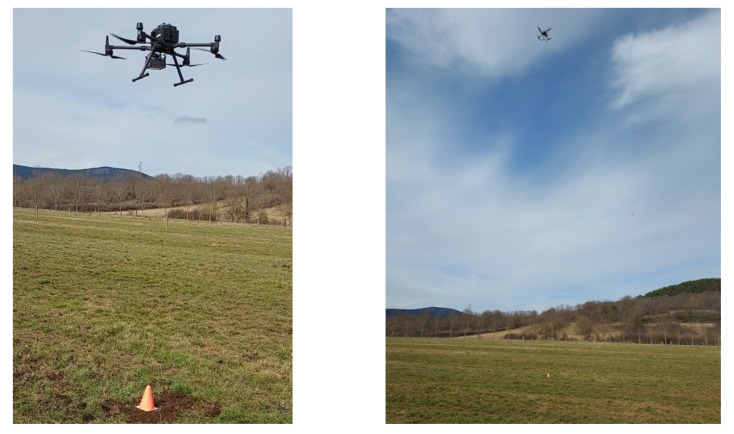
The UAV flying above the buried sensor node.

The LoRa antenna on the collector node was horizontally positioned. Figure 14 presents the flight of the UAV at different altitudes above the buried sensor node.

### 3.2. Experimental Results

#### 3.2.1. Hover Flights

**Figure 15:**
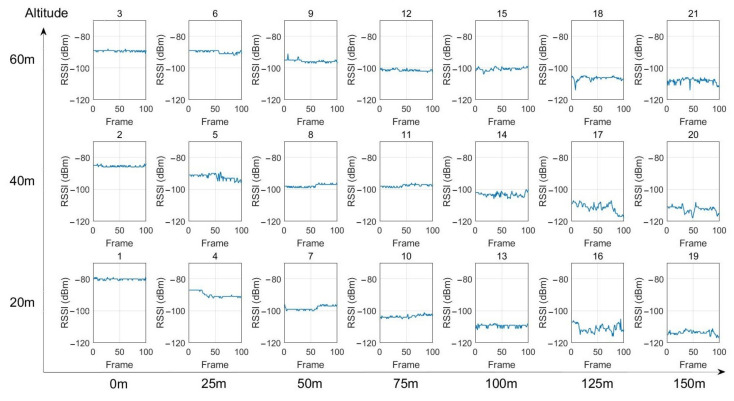
RSSI levels for each position of the UAV.

**Figure 16:**
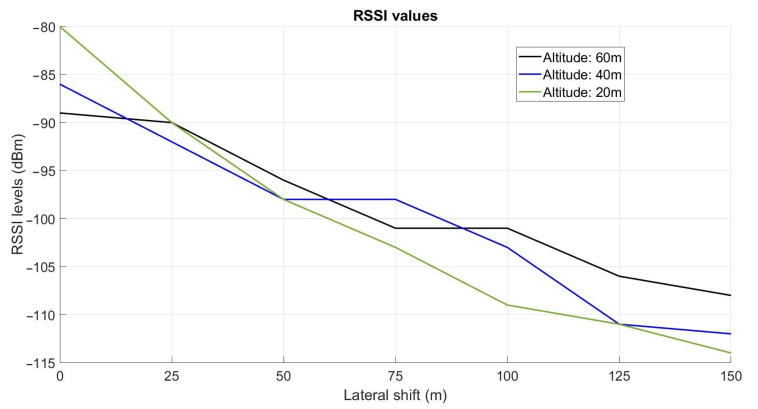
Evolution of the RSSI levels with respect to the lateral shift of the UAV.

The UAV was successively maintained in hover flight for a few seconds in each of the 21 positions defined in the experimental setup presented in Figure 9. At each position, 100 data frames were sent by the buried sensor node. The collector node received the data and measured the RSSI level. Figure 15 presents the RSSI levels obtained at each position of the UAV. These results are summarized in Table 3. The evolution of the RSSI levels with respect to the UAV’s altitude is presented in Figure 16.

From Figure 16 and Table 3, we can first observe that for a given altitude of the UAV, the RSSI value decreases with respect to the lateral distance with the nodes. For example, at 60 m altitude, the RSSI value goes from −89 dBm at the vertical of the node (0 m) to −108 dBm at 150 m. This is also the case at 40 m altitude (from −86 dBm to −112 dBm) and 20 m (from −80 dBm to −114 dBm). Next, we can observe that from the lateral distance 50 m, the RSSI values are lower at low altitude than at high altitude. For example, for a lateral distance of 100 m, the RSSI value at 20 m altitude is −109 dBm, whereas, at 60 m altitude, this value is only −101 dBm. The impact of the soil can explain that phenomenon. This result is particularly interesting as that means the UAV must fly at a high altitude to have a good signal reception when it is far from the node (more than 50 m). The high altitude enables moreover to perform safe flights as there are fewer obstacles at high altitudes than close to the soil. Near the vertical of the node (lateral distance 25 m), the RSSI values are similar at the three altitudes. At the vertical of the node (0 m), the better signal reception is at low altitude (−80 dBm at 20 m) instead of high altitude (−89 dBm at 60 m).

#### 3.2.2. Dynamic Flights

**Figure 17:**
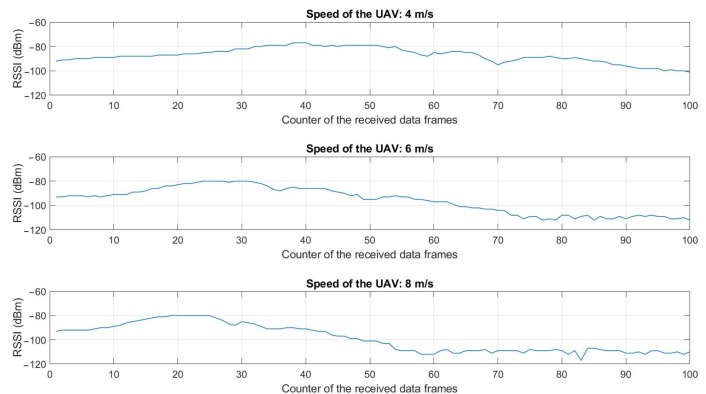
RSSI levels measured by the collector node when the UAV was flying along the trajectory of 200 m at respectively 4 m/s, 6 m/s and 8 m/s. 100 data frames were collected at each experiment.

The experimental setup defined in Figure 10 was followed for the dynamical tests. Figure 17 presents the measured RSSI levels when the UAV was flying at 40 m altitude and at respectively the speed of 4 m/s, 6 m/s, and 8 m/s.

For each experiment, the maximum RSSI value (about −80 dBm) corresponds to the time the UAV flies at the vertical of the node. At 4 m/s, this moment arrives slowly (during the reception of the data frame 40) as the UAV flies slowly. At 6 m/s, it is during the reception of the data frame 30 and at 8 m/s at the data frame 20. In this last case, the UAV arrived at the end of its 200 m trajectory and stayed in hover flight. The data continued to be stored (from the data frame 60 to 100). These results highlight the possibility of collecting the data frames when the UAV is moving, but also the necessity to properly define the speed of the UAV in the trajectory planning task with respect to the time required to retrieve all the data memorized in the considered sensor node. In our case, the transmit time of a single data frame of 25 bytes was 45 ms as presented in Table 2. As a delay of 300 ms was added between each transmission, it took about 35 s to transmit and retrieve the set of 100 data frames.

### 3.3. Trajectory Planning with GA and ACO Algorithms

**Figure 18:**
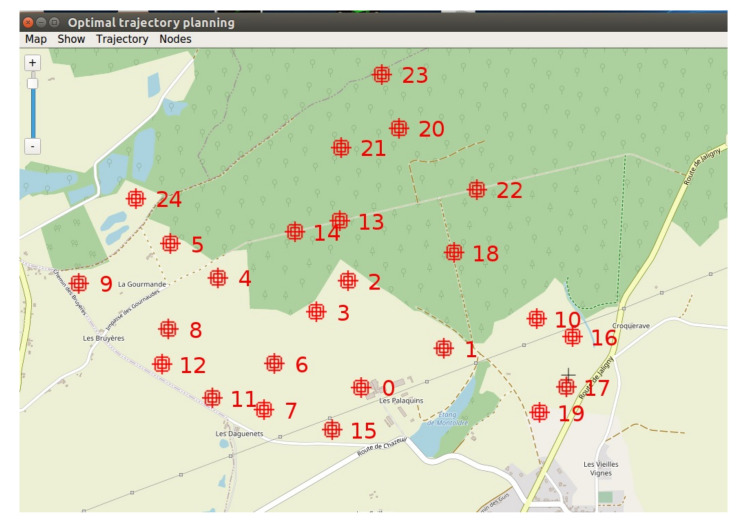
Development of a trajectory planning software in C++, displayed with OpenStreetMap. 25 buried nodes are distributed in the environment. Beforehand the use of evolutionary algorithms, these coordinates are converted into metrics coordinates using the french transformation Lambert.

In the case of numerous buried sensor nodes to be visited, a trajectory planning software was developed to define the trajectory of the UAV, see Figure 18.

**Figure 19:**
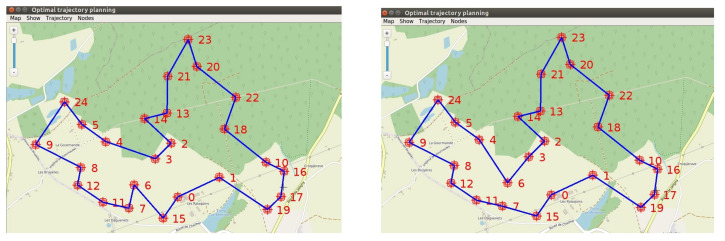
After 1000 iterations, GA found a trajectory of 8324 m (left figure) and ACO a trajectory of 8326 m (right figure). The parameters for ACO were 50 ants, α=β=1 (weights) and ρ=0.05 (evaporation). The differences between the trajectories are located near the nodes 4, 6, and 3.

**Figure 20:**
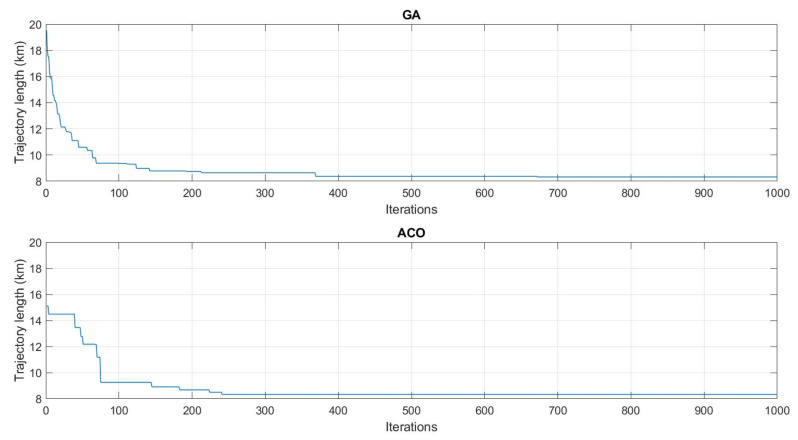
Evolution of the GA and ACO algorithms. GA converges from 19,537 m (first iteration) to 8324 m. ACO converges from 15,106 m (first iteration) to 8326 m.

The GA and ACO were implemented and tested assuming a set of 25 buried sensor nodes deployed around our experimental farm (INRAE, Montoldre, France). The coordinates of the nodes are given in WGS84 in Table A1 in Appendix A. The results are presented in Figure 19. After 1000 iterations for both algorithms, the GA and ACO delivered similar trajectories, respectively, of length 8324 m and 8326 m. We can notice that if the UAV flies at the speed of 8 m/s, it takes about 17 min to follow this trajectory, which is in accordance with its flight autonomy (about 40 min). That requires defining the number of data frames well to be collected at this speed or planning several trajectories. The execution time of the GA was faster (2.406 s) than the ACO (24.158 s). Figure 20 presents the evolution of the algorithms. The progressions of the algorithms are similar. After 100 iterations, they have already found a short trajectory, and rapidly stabilize afterwards.

## 4. Results Discussion

The objective of this study was to experimentally demonstrate the possibility of retrieving data frames of buried sensor nodes operating in LoRa from a collector node embedded in a UAV. This study is distinguished from other work by the report of experimental results combining WUSN and UAV technologies (see the summaries of the work cited in this paper in Table A2). Table 4 summarizes the setup data of the developed system.

The impact of the UAV’s lateral position and altitude on the RSSI levels was first highlighted from experimentations performed in hovering mode. 21 targeted positions were defined, composed of 3 altitudes (20 m, 40 m, 60 m) and 7 lateral distances (0 m, 25 m, 50 m, 75 m, 100 m, 125 m, 150 m). The results showed that, for a given altitude, the RSSI value decreases progressively as the UAV is positioned away from the buried sensor node. For example, at 60 m altitude, the RSSI value goes from −89 dBm at the vertical of the node to −108 dBm at 150 m. Moreover, the results clearly showed the benefit in terms of RSSI signal to fly at a relatively high altitude when the UAV is far from the node. In fact, from the lateral distance of 50 m, the RSSI values are higher at high altitude than at low altitudes. For example, at the lateral distance of 100 m, the RSSI value at 60 m altitudes is −101 dBm, whereas, at 20 m altitude, the RSSI value is −109 dBm. The impact of the soil on the signal propagation can explain that phenomenon. On the contrary, near the vertical of the node, the results showed that it is preferable to fly at low altitude.

The data collection process can also be performed when the UAV is moving. Experiments were performed at different speeds, from 4 m/s to 8 m/s. 100 data frames, memorized in the buried sensor node, were transmitted, with a delay of 300 ms between each data frame transmission. The data frames were correctly collected at the three velocities, highlighting the possibility of performing this task dynamically. However, that dynamic approach requires carefully determining the time to collect the data frames, depending on numerous factors. One of them is obviously the number of data to be collected from a buried sensor node. The parameters of the LoRa communication, in particular the SF, BW, and CR, also impact the transmit time of a data frame. If these factors are known, the time to collect the data can be previously estimated to adapt the speed and trajectory of the UAV accordingly.

Finally, two evolutionary algorithms were implemented to plan the trajectory of the UAV in case of numerous buried sensor nodes to be visited. Considering a set of 25 buried sensor nodes distributed in the environment, similar results were obtained in terms of trajectory planning after 1000 iterations with a genetic algorithm and an ant colony algorithm. The execution time of the genetic algorithm was, however, faster than with the ant colony algorithm, respectively 2.4 s and 24.2 s. Table 5 gives an overview of the main results of the experimentations.

## 5. Conclusions

This paper considers natural environments where the network coverage by GSM or WiFi is unavailable. It experimentally demonstrates the possibility of collecting information from buried sensor nodes by means of a UAV flying over the interest zones and storing the data frames through an embedded collector node. The data frames are transmitted via a LoRa communication and delivered at the end of the flight to a gateway via a ZigBee communication. The experiments performed in hover mode enabled us to highlight the best position for the UAV to collect the data with the maximum RSSI signal. The dynamic experiments demonstrated the possibility of performing the data collection task while the UAV is moving, requiring adapting the speed of the UAV accordingly. In case of several sensor nodes distributed in the environment, two evolutionary algorithms were implemented to define the best route in terms of Euclidian distance to minimize the flight time of the UAV.

From the user’s point of view, the most time-consuming task of such an approach will be to bury the sensor nodes in the environment. Once this work will be performed, the nodes could stay underground for a long time, ideally one year, without interventions. In the initial calibration, the knowledge of the GPS position of each buried sensor node is required to program the flight plan of the UAV. Table 6 presents a list of advantages and disadvantages of an automated solution compared to a manual one. The automated solution requires the purchase of a UAV and needs a drone pilot to configure and monitor the UAV. The flight of the UAV is also constrained by the weather conditions (e.g., a flight can be postponed in case of too heavy rainfalls or too strong winds, the battery are sensible to low temperatures). As opposed to the autonomous approach, the manual solution would require regular displacements of an operator on the sites. These displacements can take a long time and be tedious and costly over time.

## 6. Future Work

Our future work will concentrate on the improvement of the UAV’s trajectory planning algorithm with the consideration of additional constraints. In particular, the definition of priority will be added on each waypoint as some data can be more urgent to collect on some buried sensor nodes than others. Moreover, clusters of waypoints will be defined as the UAV has not to be at the vertical of a buried sensor node to collect the data. The experiments reported in this paper showed, in fact, the possibility to horizontally shift the UAV of 150 m from the buried node and collect the data despite this shift (−108 dBm at 150 m and 60 m altitude). Intersections of coverage ranges can therefore be taken into account. In addition, as previously mentioned, it would be interesting to predict the time necessary for the data collection process with respect to different factors (e.g., the date of the last data collection to have the number of data frames to collect, LoRa parameters) and to predict the communication range of each buried sensor nodes with respect to the burial depth and various environmental factors (e.g., soil moisture, soil composition, compaction) to adapt the UAV’s flight altitude. Some situations may also require several flights to collect the data of all the nodes as the UAV has the autonomy of flight limited. All these aspects require the development of optimization algorithms and adapting the trajectories of UAV accordingly.

In parallel, some experiments will be carried out with sensor nodes buried more deeply. This depth will be a function of the area where the nodes will be positioned (e.g., edge of the field, under a circulation area, in the middle of a field with farm activities). In addition, the analysis of the quality of the communication link between the buried sensor nodes and the collector node will be carried out. To this aim, the percentage of the lost data frames will be studied with respect to the quality of the LoRa communication link (RSSI levels). It would be necessary to improve the protocol of the bi-directional communication link between the buried sensor nodes and the collector node. All these developments, combining WUSNs and UAVs technologies, aim to open new perspectives in the area of environmental monitoring. 

## Figures and Tables

**Table 1:** Structure of a data frame sent by a buried sensor node (25 bytes).

Name	Description
SensorNodeID	Sensor node identifier
Counter	Auto increment of the frame
TimestampMeasure	Measurement Timestamp (UTC time)
NodeBatt	Battery voltage of the node
Permittivity	Data of the probe SMT100
Humidity	Data of the probe SMT100
Temperature	Data of the probe SMT100

**Table 2:** RF transmit time of a data frame of 25 bytes with respect to SF (BW = 125 KHz, CR = 4/5). Values obtained from Semtech’s datasheets [40].

	SF7	SF8	SF9	SF10	SF11	SF12
RF Tx time (ms)	45	80	160	280	561	958

**Table 3:** Mean of the RSSI values (dBm) at each position (altitude, lateral distance).

*60 m*	−89	−90	−96	−101	−101	−106	−108
*40 m*	−86	−92	−98	−98	−103	−111	−112
*20 m*	−80	−90	−98	−103	−109	−111	−114
	*0 m*	*25 m*	*50 m*	*75 m*	*100 m*	*125 m*	*150 m*

**Table 4:** Summary of the setup data.

Configuration of the buried sensor nodes	- Parameters (LoRa): SF7, CR 4/5, BW 125 kHz - Transmit power (LoRa): +14 dBm/25 mW - Frequency (LoRa): 868 MHz - Antenna orientation (LoRa): vertical - Depth: 15 cm - Data frame: 25 bytes (ID, counter, timestamp, battery voltage, permittivity, humidity, temperature) - Trigger: send a set of 100 data frames with a delay of 300 ms between each transmission
Configuration of the collector node	- Parameters (LoRa): SF7, CR 4/5, BW 125 kHz - Frequency (LoRa): 868 MHz - Antenna orientation (LoRa): horizontal - End device (ZigBee-Pro): 2.4 GHz
Configuration of the gateway	- Coordinator (ZigBee-Pro): 2.4 GHz
UAV	- Hover flight (21 positions): 3 altitudes (20 m, 40 m, 60 m) and 7 lateral distances (0, 25 m, 50 m, 75 m, 100 m, 125 m, 150 m) - Dynamic mode: 1 altitude (40 m), 3 speeds (4 m/s, 6 m/s, 8 m/s)
Configuration of the trajectory planning algorithms	- GA: initial population with 100 routes, groups of 4 routes, operators = reverse, shift, swap, 1000 iterations - ACO: 50 ants, evaporation 5%, 1000 iterations

**Table 5:** Summary of the experimental results.

Experiments in hover mode	- At the vertical of the node, the signal reception is better at low altitude - Near the vertical of the node, the signal reception is similar at the three altitudes - Far from the node, the signal reception is better at high altitude
The data frames are correctly collected at the three velocities	- UAV velocity will have to be adapted to the time required to retrieve all the data memorized in the buried sensor node
Results of trajectory planning	- Similar results obtained for GA and ACO in terms of trajectory planning - Execution time higher for ACO than GA

**Table 6:** Advantages and disadvantages of the automated solution compared to the manual solution.

	Advantages	Disadvantages
Automated data collection by means of a UAV	- Possibility of data collection at high-frequency - Displacement on site not needed - Possibility to go within difficult access areas - Time-saving - Possibility of data collection in areas not covered by wireless networks	- Dependent of weather conditions - Require a drone pilot
Data collection by a human operator	- Simplicity (no special skills needed) - Possibility of data collection in areas not covered by wireless networks	- The operator has to go frequently to the sites to collect the data (tedious, time-consuming) - The frequency of data collection is dependent of the operator’s availability

## Data Availability

Not applicable.

## Abbreviations

The following abbreviations are used in this manuscript:

ACO	Ant Colony Optimization
GA	Genetic Algorithm
IoT	Internet of Things
RSSI	Received Signal Strength Indication
SF	Spreading Factor
TSP	Traveling Salesman Problem
UAV	Unmanned Aerial Vehicle
UG2AIR	Underground to Air
VWC	Volumetric Water Content
WSN	Wireless Sensor Network
WUSN	Wireless Underground Sensor Network

## Appendix A

**Table A1:** List of the 25 nodes used to test the optimization algorithms. The coordinates are given in the WGS84 reference system.

Node	Latitude (°)	Longitude (°)
0	46.340252	3.432973
1	46.341912	3.438121
2	46.344821	3.432128
3	46.343507	3.430192
4	46.344978	3.424024
5	46.346443	3.421086
6	46.341281	3.427551
7	46.339276	3.426908
8	46.342764	3.420956
9	46.344727	3.415366
10	46.343209	3.443890
11	46.339782	3.423708
12	46.341258	3.420548
13	46.347399	3.431639
14	46.346931	3.428845
15	46.338440	3.431160
16	46.342437	3.446153
17	46.340274	3.445739
18	46.346031	3.438756
19	46.339173	3.444083
20	46.351383	3.435313
21	46.350550	3.431701
22	46.348745	3.440153
23	46.353679	3.434268
24	46.348362	3.418943

**Table A2:** Previous research work focused on WSN, WUSN and UAV.

Ref.	Methodology	Results and Findings
[5]	- Analysis of the underground wireless channel. - Simulation and field experiments. - Operating frequency: 433 MHz	- State-of-the-art - Impacts of the burial depth, soil composition, soil moisture, reflection, refraction, multi-path fading, antenna orientation, inter-node distance.
[6]	- IOUT in precision agriculture - State-of-the-art and challenges	- Presentation of academic and commercial systems (sensors, wireless technologies, communication architectures.)
[7]	- Modelization to estimate the signal attenuation in the ground. - Laboratory experimentations - Operating frequency: 2.44 GHz	- Impact of soil depth, soil water content, soil electrical conductivity on the UG2AG communications.
[8]	- Application: urban drainage system - Actual experimentations - Comparison of LoRa and LoRaWAN in terms of packet loss (UG to AG gateway).	- Reliability of LoRa when transmitting from range-critical locations.
[9]	- WUSN in precision agriculture (center pivot for irrigation) - Field experimentations (corn field) - Operating frequency: 433 MHz	- Proof-of-concept - The growth of the crop and soil moisture affect the UG2AG communication link - Highlight research challenges
[10]	- Present the concept of WUSN. List of impacting factors, overview of potential applications and challenges.	- State-of-the-art
[11]	- WUSN to monitor landslides - Actual implementation, measurements of soil moisture, storage of the data on a Web Server.	- Proof-of-concept
[12]	- Present the concept and challenges of WUSN. - Discussion on the technological advances, compare the communication EM (electromagnetic waves radiation) and MI (magnetic induction). - Modelization of signal propagation through soil	- State-of-the-art
[13]	- Presentation and comparison of propagation models of signal propagation through soil. - Actual experiments to evaluate the models	- Propose a new propagation model to cover path loss induced by different factors (soil absorption, radiation, reflection, wavelength change)
[14]	- Present the state-of-the-art of IOUT, potential applications, and challenges. - Highlight different wireless technologies.	- State-of-the-art
[15]	- Present the state-of-the-art of IOUT, architectures, potential applications, and challenges.	- State-of-the-art
[16]	- Actual experiments - Operating frequency: 433 MHz	- Low frequencies lead to smaller signal attenuation - VWC highly impacts the underground communications - Impact of antenna orientation, burial depth, soil moisture.
[17]	- Evaluation of LoRa technology in 433 MHz and 868 MHz in different conditions for UG2AG (transmit power, burial depth, antenna in contact with the soil, antenna inclination) - Actual experiments	- Better results with 868 MHz radio modules in terms of communication range
[19]	- Test the LoRaWAN protocol for underground communication in different conditions (radio parameters, burial depth) - Actual experiments	- Tests from 10 cm to 50 cm depth. At 50 cm depth, the packet loss is lower than 1 %
[22]	- State-of-the-art in smart farming based on IoT and UAV. - Development of a platform with sensors distributed on a farm, and an UAV collecting the data (a LoRa gateway is embedded) and transmitting them to the cloud through a 4 G transceiver. - Actual experiments	- Proof-of-concept - Highlight the limited flight time of the UAV and the need to optimize the power consumption - Propose to develop a machine learning algorithm to predict environmental conditions to achieve an autonomous system
[26]	- Concept of a mobile data collector. - Present a communication architecture and protocols enabling a UAV to communicate in a bi-directional manner with a WSN - Simulations and actual experiments	- Proof-of-concept

## References

[1] National Academy of Sciences (2020). Climate Change: Evidence and Causes: Update 2020.

[2] Jawak S.D., Wankhede S.F., Luis A.J., Balakrishna K. (2022). Impact of image-processing routines on mapping glacier surface facies from svalbard and the himalayas using pixel-based methods. Remote Sens..

[3] Li J., Ma R., Cao Z., Xue K., Xiong J., Hu M., Feng X. (2022). Satellite detection of surface water extent: A review of methodology. Water.

[4] Hussain S., Lu L., Mubeen M., Nasim W., Karappannan S., Fahad S., Tariq A., Mousa B.G., Mumtaz F., Aslam M. (2022). Spatiotemporal variation in land use land cover in the response to local climate change using multispectral remote sensing data. Land.

[5] Vuran M.C., Silva A.R. (2009). Communication through soil in wireless underground sensor networks—Theory and practice. Sensor Networks—Where Theory Meets Practice.

[6] Vuran M.C., Salam A., Wong R., Irmak S. Internet of underground things: Sensing and communications on the field for precision agriculture. Proceedings of the IEEE 4th World Forum on Internet of Things (WF-IoT).

[7] Bogena H.R., Huisman J.A., Meier H., Rosenbaum U., Weuthen A. (2009). Hybrid wireless underground sensor networks: Quantification of signal attenuation in soil. Vadose Zone J..

[8] Ebi C., Schaltegger F., Rüst A., Blumensaat F. (2019). Synchronous LoRa mesh network to monitor processes in underground infrastructure. IEEE Access.

[9] Silva A.R., Vuran M.C. (CPS)2: Integration of center pivot systems with wireless underground sensor networks for autonomous precision agriculture. Proceedings of the IEEE/ACM International Conference on Cyber-Physical Systems.

[10] Akyildiz I.F., Stuntebeck E.P. (2006). Wireless underground sensor networks: Research challenges. Ad Hoc Netw..

[11] Ferreira C.B.M., Peixoto V.F., de Brito J.A.G., de Monteiro A.F.A., de Assis L.S., Henriques F.R. (2019). UnderApp: A system for remote monitoring of landslides based on wireless underground sensor networks. WTIC.

[12] Da Silva A.R., Moghaddam M., Liu M. (2014). The future of wireless underground sensing networks considering physical layer aspects. Art Wirel. Sens. Netw..

[13] Huang H., Shi J., Wang F., Zhang D. (2020). Theoretical and experimental studies on the signal propagation in soil for wireless underground sensor networks. Sensors.

[14] Saeed N., Alouini M.S., Al-Naffouri T. (2019). Towards the Internet of Underground Things: A systematic survey. IEEE Commun. Surv. Tutorials.

[15] Salam A., Raza U. (2020). Current advances in Internet of Underground Things. Signals in the Soil.

[16] Silva A.R., Vuran M.C. Empirical evaluation of wireless underground-to-underground communication in wireless underground sensor networks. Proceedings of the IEEE 5th International Conference on Distributed Computing in Sensor Systems.

[17] Moiroux-Arvis L., Cariou C., Chanet J.P. (2022). Evaluation of LoRa technology in 433-MHz and 868-MHz for underground to aboveground data transmission. Comput. Electron. Agric..

[18] Augustin A., Yi J., Clausen T., Townsley W.M. (2016). A study of LoRa: Long range & low power networks for the Internet of Things. Sensors.

[19] Gineprini M., Parrino S., Peruzzi G., Pozzebon A. LoRaWAN performances for underground to aboveground data transmission. Proceedings of the IEEE International Instrumentation and Measurement Technology Conference.

[20] LoRa Alliance LoRaWAN 1.0.3 Specification; 2018; pp. 1–72. https://lora-alliance.org/resource_hub/lorawan-specification-v1-0-3/.

[21] Mukhamediev R.I., Symagulov A., Kuchin Y., Zaitseva E., Bekbotayeva A., Yakunin K., Assanov L., Levashenko V., Popova Y., Akzhalova A. (2021). Review of some applications of unmanned aerial vehicles technology in the resource-rich country. Appl. Sci..

[22] Almalki F.A., Soufiene B.O., Alsamhi S.H., Sakli H. (2021). A Low-Cost Platform for Environmental Smart Farming Monitoring System Based on IoT and UAVs. Sustainability.

[23] Gupta A., Afrin T., Scully E., Yodo N. (2021). Advances of UAVs toward future transportation: The state-of-the-art, challenges, and opportunities. Future Transp..

[24] Chandrasekharan S., Gomez K., Al-Hourani A., Kandeepan S. (2016). Designing and implementing future aerial communication networks. IEEE Commun. Mag..

[25] Zeng Y., Zhang R., Lim T.J. (2016). Wireless communications with unmanned aerial vehicles: Opportunities and challenges. IEEE Commun. Mag..

[26] Sayyed A., Araujo G.M., Bodanese J.P., Becker L.B. (2015). Dual-stack single-radio communication architecture for UAV acting as a mobile node to collect data in WSNs. Sensors.

[27] Mozaffari M., Saad W., Bennis M., Nam Y.H., Debbah M. (2019). A tutorial on UAVs for wireless networks: Applications, challenges, and open problems. IEEE Commun. Surv. Tutor..

[28] Samir M., Sharafeddine S., Assi C.M., Nguyen T.M., Ghrayeb A. (2019). UAV trajectory planning for data collection from time-constrained IoT devices. IEEE Trans. Wirel. Commun..

[29] Yang X., Fu S., Wu B., Zhang M. A survey of key issues in UAV data collection in the internet of things. Proceedings of the IEEE Int Conf on Dependable, Autonomic and Secure Computing Int Conf on Pervasive Intelligence and Computing, Int Conf on Cloud and Big Data Computing, Int Conf on Cyber Science and Technology Congress.

[30] Popescu D., Stoican F., Stamatescu G., Ichim L., Dragana C. (2020). Advanced UAV–WSN System for Intelligent Monitoring in Precision Agriculture. Sensors.

[31] Popescu D., Dragana C., Stoican F., Ichim L., Stamatescu G. (2018). A collaborative UAV-WSN network for monitoring large areas. Sensors.

[32] Gong J., Chang T.S., Shen C., Chen X. (2018). Flight time minimization of UAV for data collection over wireless sensor networks. IEEE J. Sel. Areas Commun..

[33] Chen J., Dai Z., Chen Z. (2019). Development of radio-frequency sensor wake-up with unmanned aerial vehicles as an aerial gateway. Sensors.

[34] Castellanos G., Deruyck M., Martens L., Joseph W. (2020). System assessment of WUSN using NB-IoT UAV-aided networks in potato crops. IEEE Access.

[35] Muthu Ramya C., Shanmugaraj M., Prabakaran R. Study on ZigBee technology. Proceedings of the 3rd International conference on Electronics Computer Technology.

[36] Qu F., Yu W., Xiao K., Liu C., Liu W. (2022). Trajectory generation and optimization using the mutual learning and adaptive ant colony algorithm in uneven environments. Appl. Sci..

[37] Alhanjouri M.A. (2017). Optimization techniques for solving Travelling Salesman Problem. Int. J. Adv. Res. Comput. Sci. Softw. Eng..

[38] Cariou C., Gobor Z. (2018). Trajectory planning for robotic maintenance of pasture based on approximation algorithms. Biosyst. Eng..

[39] Dorigo M., Stützle T. (2004). Ant Colony Optimization.

[40] (2020). LoRa SX1276/77/78/79 Datasheet, Rev. 7. Semtech. http://www.semtech.com/.

